# A Novel Earphone Type Sensor for Measuring Mealtime: Consideration of the Method to Distinguish between Running and Meals

**DOI:** 10.3390/s17020252

**Published:** 2017-01-27

**Authors:** Kazuhiro Taniguchi, Hikaru Chiaki, Mami Kurosawa, Atsushi Nishikawa

**Affiliations:** 1 Graduate School of Information Sciences, Hiroshima City University, 3-4-1 Ozukahigashi, Asaminami-ku, Hiroshima 731-3194, Japan; hikaru-chiaki@robotics.info.hiroshima-cu.ac.jp; 2 Graduate School of Science and Technology, Shinshu University, 3-15-1, Tokida, Ueda, Nagano 386-8567, Japan; 15fm314h@shinshu-u.ac.jp; 3 Faculty of Textile Science and Technology, Shinshu University, 3-15-1, Tokida, Ueda, Nagano 386-8567, Japan; nishikawa@shinshu-u.ac.jp

**Keywords:** wearable device, ear canal, meal time estimation, body vibration on running, dietary support

## Abstract

In this study, we describe a technique for estimating meal times using an earphone-type wearable sensor. A small optical sensor composed of a light-emitting diode and phototransistor is inserted into the ear hole of a user and estimates the meal times of the user from the time variations in the amount of light received. This is achieved by emitting light toward the inside of the ear canal and receiving light reflected back from the ear canal. This proposed technique allowed “meals” to be differentiated from having conversations, sneezing, walking, ascending and descending stairs, operating a computer, and using a smartphone. Conventional devices worn on the head of users and that measure food intake can vibrate during running as the body is jolted more violently than during walking; this can result in the misidentification of running as eating by these devices. To solve this problem, we used two of our sensors simultaneously: one in the left ear and one in the right ear. This was based on our finding that measurements from the left and right ear canals have a strong correlation during running but no correlation during eating. This allows running and eating to be distinguished based on correlation coefficients, which can reduce misidentification. Moreover, by using an optical sensor composed of a semiconductor, a small and lightweight device can be created. This measurement technique can also measure body motion associated with running, and the data obtained from the optical sensor inserted into the ear can be used to support a healthy lifestyle regarding both eating and exercise.

## 1. Background and Objective

The use of wearable devices is growing in popularity in the field of healthcare [1,2,3,4,5,6,7,8,9,10,11,12,13,14,15,16]. Wristwatch-type wearable devices are particularly widespread and estimate the amount of exercise by measuring body temperature and heart rate, thereby supporting a user’s diet by presenting these estimates. In dietary support, the use of measured results for meals is just as important as support that uses measured results for the amount of exercise. Calorie control is the main form of support for meals; however, support using meal time management has recently received growing attention. Irregular meal times and eating before bedtime have been found to be some of the factors that promote the onset and progression of lifestyle-related diseases [17]. However, support for ordinary individuals using meal time management remains limited due to some challenges, including the lack of awareness among society regarding the importance of meal times and the lack of wearable devices that can easily and automatically estimate meal times without interfering with lifestyle.

Recently, measurements of food intake have been performed using eyeglass-type wearable devices [18,19,20]. Eyeglass-type devices use optical, strain, and other sensors to measure food intake. Strain sensors require close contact between the sensor and skin during measurement, whereas optical sensors can take measurements without bringing the sensor in close contact with the skin. Thus, optical sensors have a greater advantage over strain sensors as they are minimally impacted by the shape and amount hair growth on the surface of a user’s skin. Moreover, studies that have used strain sensors have been able to differentiate walking from eating by combining strain sensors with accelerometers. In eyeglass-type devices, the vibration of the devices themselves during walking affects the measured data; however, these measured values obtained from walking can be removed because they are smaller than the values measured during chewing. However, violent body motion (e.g., during running) also increases the vibration of the device itself, which can increase the effect on the values measured by this vibration, making it difficult to remove. A device can be fixed to a user’s head to minimize vibrations; however, this is more uncomfortable for a user. Therefore, vibrations of a device due to running are a difficult issue to overcome with eyeglass-type wearable devices.

**Figure 1:**
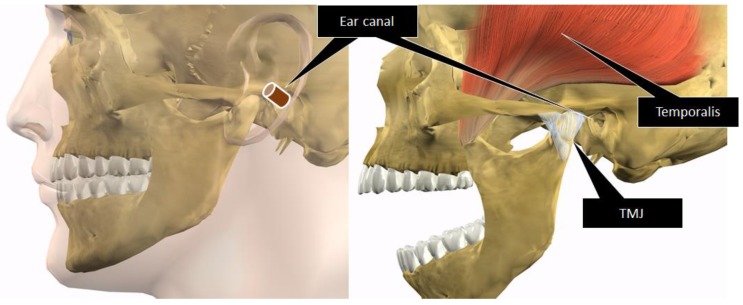
Anatomical positional relationship between the ear canal, temporal muscle (temporalis), and temporomandibular joint (TMJ). Chewing occurs by moving the TMJ and the temporal muscle. The ear canal is anatomically close to the TMJ and temporal muscle and is susceptible to mechanical force exerted by the TMJ and temporal muscle.

The objective of our study was to develop a wearable device capable of measuring food intake and differentiating eating from running. In this paper, we describe a technique for estimating meal times as the first stage in reporting these research results. The measurements of food intake by eyeglass-type devices use a sensor to measure the “movement of the masticatory muscles, including mainly the temporal muscle (temporalis)” and “movement of the skin in the temporal region resulting from the movement of the temporomandibular joint (TMJ)”. We discussed the measurements of chewing from the relationship between the anatomical position of the temporal muscle and TMJ in humans, as shown in Figure 1, and revealed that changes to the shape of the ear canal during chewing are useful for measuring food intake [21]. While the shape of the ear canal also changes during facial expressions [22], chewing tends to repeatedly change the ear canal shape, which makes it possible to distinguish chewing from facial expressions. In this paper, we describe a technique for estimating the meal times of users based on temporal changes in the amount of light received by a small optical sensor. The sensor is composed of a light-emitting diode (LED) and phototransistor inserted into the ear hole which emits light toward the inside of the ear canal and receives light reflected back from the ear canal to measure the change in the amount of light. We also proposed a method for differentiating eating from running.

## 2. Materials and Methods

### 2.1. Measurement Principle and Sensor Prototype

**Figure 2:**
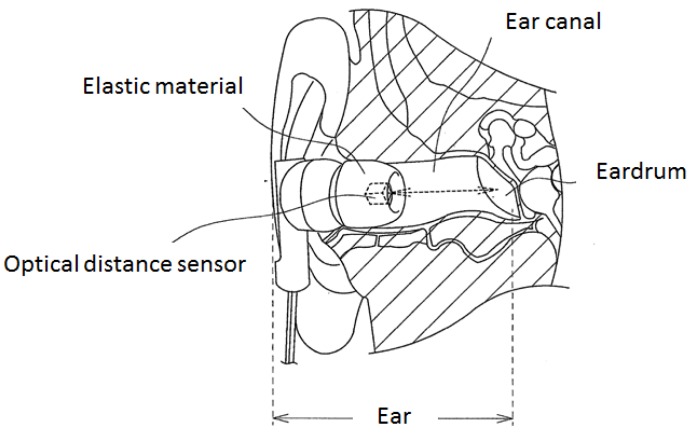
Measurement principle for changes in the shape of the ear canal. The earphone-type sensor receives the light emitted from the optical distance sensor that is reflected back by the eardrum and ear canal. During chewing, the shape of the ear canal changes, which alters the distance between the optical distance sensor and the eardrum and ear canal. The amount of light received changes over time in association with this change in distance.

Figure 2 presents a schematic diagram of a sensor designed to estimate meal times. The sensor in Figure 2 measures movement in the ear associated with eating. Using this method, movement in the ear is measured by light. Using light, eating can be measured without the sensor irritating the sensitive ear. Eating measurements are done by inserting an earphone-type sensor into the ear hole (ear canal), as shown in Figure 2. This earphone-type sensor has a built-in optical distance sensor that is composed of a light-emitting portion (LED) and a light-receiving portion (phototransistor). The earphone-type sensor receives the light emitted from the optical distance sensor that is reflected back by the eardrum and ear canal. During chewing, the shape of the ear canal changes, which alters the distance between the optical distance sensor and the eardrum and ear canal. The amount of light received changes over time in association with this change in distance. In this study, we estimated meal times by analyzing temporal changes in the amount of light. The LED of the optical distance sensor used in the earphone-type sensor emits infrared light at a wavelength of 940 nm, which is rare in the natural world (sunlight does not contain a substantial amount of this wavelength of infrared light).

The optical distance sensor within the earphone-type sensor takes measurements within the closed space of the ear canal, which limits the effect of light from outside the body and does not restrict the environments in which a user can employ the sensor (it can be used both indoors and outdoors, during summer and winter, and in the day and at night). While the earphone-type sensor only vibrates slightly as a result of intense movement (e.g., running), the sensor is securely surrounded by the ear canal, which minimizes vibrations compared with eyeglass-type devices. The earphone-type sensor is also fitted with an optical distance sensor inside a casing that is the same as that widely used in conventional inner ear-type earphones, which allows the earphone-type sensor to be worn as easily as a conventional earphone.

**Figure 3:**
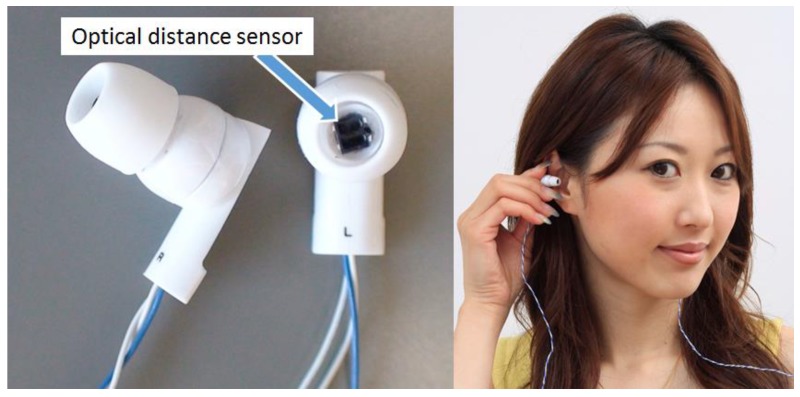
Sensor prototype. The earphone-type sensor has the same shape as a conventional inner ear-type earphone. The sensor is worn and used in both ears.

**Figure 4:**
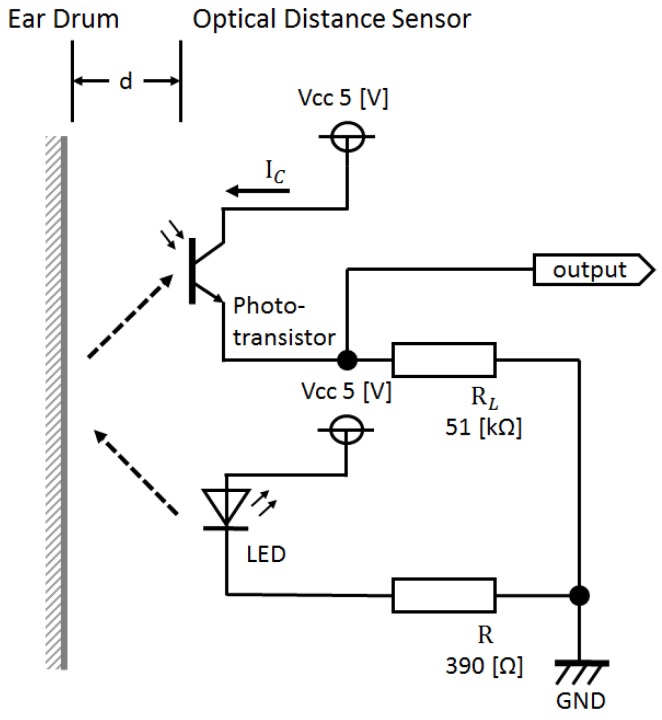
Electronic circuit around the sensor. From the circuit, it is evident that the reflected light changes based on the distance d between the phototransistor and vicinity of the eardrum, which causes the collector current *I*_C_ of the phototransistor to change in response to fluctuations in the shape of the ear canal associated with chewing. The change in the collector current *I*_C_ obtained here is converted into a change in the voltage of the resistor *R*_L_. This change in voltage is considered as the output voltage of the sensor.

Figure 3 shows the outer appearance of the prototype earphone-type sensor, while Figure 4 is a representation of its electronic circuit. As shown in Figure 3, the earphone-type sensor has the same shape as a conventional inner ear-type earphone, and is worn and used in both ears. This prototype optical distance sensor uses a KODENSHI SG-105 photo reflector (KODENSHI TK corp., Tokyo, Japan, a Fairchild QRE1113 Reflective Object Sensor can also be used.). As shown in Figure 3, infrared light from an LED is in the vicinity of the eardrum and is received by a phototransistor by placing an optical distance sensor in the same casing as a conventional inner ear-type earphone. In the circuit presented in Figure 4, it is evident that the reflected light changes based on the distance *d* between the phototransistor and vicinity of the eardrum, which causes the collector current *I*_C_ of the phototransistor to change in response to fluctuations in the shape of the ear canal associated with chewing. The change in the collector current *I*_C_ obtained here is converted into a change in the voltage of the resistor *R*_L_. This change in voltage is considered as the output voltage of the sensor.

### 2.2. Meal Time Estimation Algorithm

**Figure 5:**
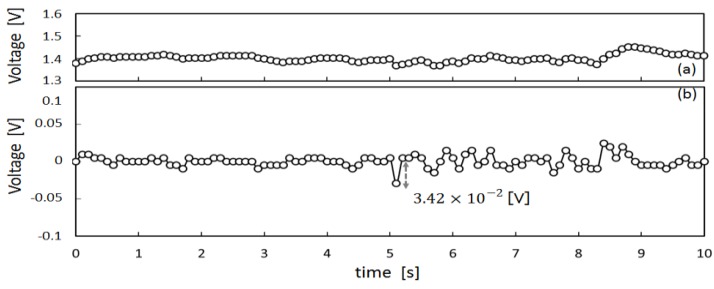
Ear-canal movement of conversation: (**a**) shows the measured values for movement of the ear canal during conversation; (**b**) shows the amount of change in these measured values. The amount of change was obtained by subtracting the immediately anteceding measured value (100 ms before obtaining the measurements because measurements are performed at 10 Hz) from the current measured value.

**Figure 6:**
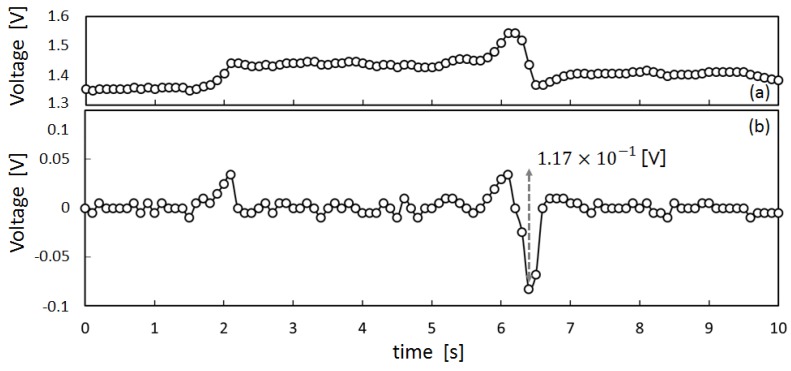
Ear-canal movement of yawn: (**a**) shows the movement of the ear canal during yawning, while (**b**) shows the corresponding amount of change.

**Figure 7:**
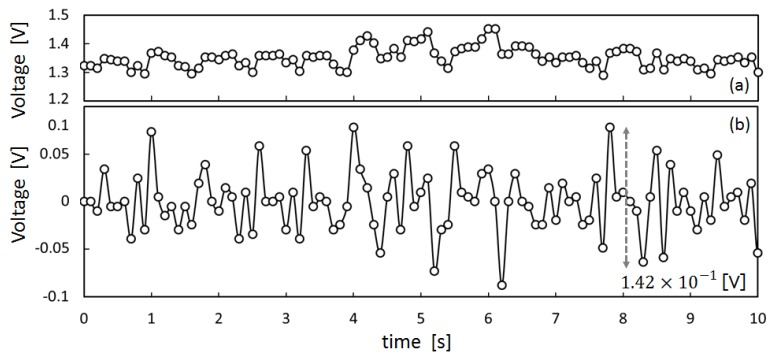
Ear-canal movement of chewing: (**a**) shows the movement of the ear canal during gum chewing, while (**b**) shows the corresponding amount of change.

To obtain preliminary data to design a meal time estimation algorithm, we measured the movement of the ear canal of subject X by means of the earphone-type sensor shown in Figure 4. The measured results are presented in Figure 5, Figure 6 and Figure 7. Figure 5a shows the measured values for movement of the ear canal during conversations, and Figure 5b presents the amount of change in these measured values. The amount of change was obtained by subtracting the immediately anteceding measured value (100 ms prior to obtaining measurements because measurements are performed at 10 Hz) from the current measured value. Figure 6a shows the movement of the ear canal during yawning, while Figure 6b shows the corresponding amount of change. Figure 7a presents the movement of the ear canal while chewing gum, while Figure 7b shows the corresponding amount of change. Figure 5b and Figure 7b show that the amount of change is $3.42\times 10^{-2}$ V during conversations and $1.42\times 10^{-1}$ V while chewing gum. A comparison of the amount of change during conversations and chewing gum reveals that the amount of change is greater during chewing gum; this is because the movement of the ear canal is greater. Furthermore, Figure 6 shows that the amount of change during yawning is $1.17\times 10^{-1}$ V, which is similar to the amount of change during chewing and greater than the amount of change during conversations. Yawning and chewing gum are similar in that they involve greater movement of the ear canal than conversation. However, a comparison of Figure 6 and Figure 7 reveals that chewing gum is characterized by a large “continuous” change.

In this study, we used the fact that shape changes to the ear canal persist longer than changes due to facial expressions during chewing to estimate meal times. We aimed for an accuracy of within five minutes and used the newly defined “Meal Quality Feature (MQF)” for meal time estimation. The MQF is determined by obtaining the absolute value of the amount of change based on shape changes to the ear canal and calculating the sum of the mean and standard deviation of this absolute value within a certain period. During eating, the ear canal changes in shape considerably more than during facial expressions as a result of chewing. Because this change is continuous, the MQF of the time spent eating is greater than the MQF of the time spent not eating. A threshold is introduced to determine the size relationship of the calculated MQF. Shape changes to the ear canal differ among individuals, which results in a different MQF value being calculated for each subject. In this study, a threshold that differs depending on the subject was introduced instead of a uniquely determined threshold. The threshold was 50% of the difference between the maximum (max) and minimum (min) MQF calculated for each subject. The time during which the MQF continues to remain above the threshold is the meal time. The MQF is calculated by grouping the measured values into five-minute intervals, such that the estimated meal time includes approximately five minutes of error. The details of the meal time estimation algorithm are written below. Furthermore, while the sensor is worn and used in both ears, this algorithm is capable of estimating meal times using only the sensor information obtained from either the left or right ear.

Step 1. The mealtime estimation interval *t*_interval_ is set at 5 min (i.e., 300 s). The sampling frequency *f* of the 10-bit analog to digital (AD) converter used in the measurements is set at *f* = 10 Hz. This is because chewing involves a constant cycle of repetitive, alternate contractions of the mouth’s opening and closing muscles within the frequency range of 1.1–1.7 Hz [23].
Step 2. The sensor wearing time *t*_wear_ is at first decided, and then shape changes to the ear canal are measured for *t*_wear_ (seconds) by the sensor shown in Figure 3 and Figure 4. The measured values are converted to measured data using ***a*** = {*a*_1_, *a*_2_, …, *a_n_*} (unit: V), where *n* represents the number of data measurements, and *n* = *t*_wear_ *× f*. For example, when a subject wears the sensor for two hours a day (*t*_wear_ = 7200 s), *n* = 7200 *×* 10 = 72,000.
Step 3. Outlying values included among the measured data ***a*** are found. The overall mean of the measured data ***a*** is determined. Then 130% and 70% of this overall mean are set as the upper limit *S*_u_ and lower limit *S*_l_, respectively, to establish a tolerance range [*S*_l_, *S*_u_]. Considering measured data *a_i_* (*i* = 1, 2, …, *n*) outside the range [*S*_l_, *S*_u_], all values within one second before and after this range {*a_i −_*_5_, …, *a_i_*, …, *a_i_*_+ 5_} are set as outlying values. The identification of outlying values helped to eliminate sensor signals with an amplitude greater than that of chewing, which were observed during the removal of the earphone-type sensor and readjustment of its position. It also allowed for the elimination of signals with an amplitude smaller than that of chewing, which were observed during vibration of the sensor cable.
Step 4. The absolute value *c_i_* of the amount of change in the shape of the ear canal is determined using Equation (1) and is set as the data for the amount of change ***c*** = {*c*_1_, *c*_2_, …, *c_i_*, …, *c_n_*}.
  (1) $$c_{i}=\{\begin{array}{c} \left|a_{i+1}-a_{i}\right|\;\mathrm{if}\;\mathrm{neither}\;a_{i}\;\mathrm{nor}\;a_{i+1}\;\mathrm{is}\;\mathrm{an}\;\mathrm{outlying}\;\mathrm{value}\;\\ “\mathrm{outlying}\;\mathrm{value}”\;\mathrm{otherwise}\; \end{array}$$
Step 5. The calculated data ***c*** = {*c*_1_, *c*_2_, …, *c_n_*} is divided into {***b***_1_, ***b***_2_, …, ***b****_j_*, …, ***b****_p_*}, where *p* = *t*_wear_/*t*_interval_, and the number of elements included in each ***b****_j_* (called *n′*) is given by *n′* = *n*/*p*. In this case, ***b****_j_* = {*c*_min__(*j*)_, *c*_min__(*j*)+1_, …, *c*_max__(*j*)−1_, *c*_max__(*j*)_}, where min(*j*) = (*j* *−* 1) *× n′*, and max(*j*) = *j × n′* (*j* = 1, 2, …, *p*).
    For example, when a subject wears the sensor for two hours per day (*t*_wear_ = 7200 s), *p* = 7200/300 = 24, and *n′* = 72,000/24 = 3000. As a result, the calculated data ***c*** = {*c*_1_, *c*_2_, …, *c*_72,000_} is divided into {***b***_1_, ***b***_2_, …, ***b***_24_}, where ***b***_1_ = {*c*_1_, *c*_2_, …, *c*_3000_}, ***b***_2_ = {*c*_3001_, *c*_3002_, …, *c*_6000_}, . . ., and ***b***_24_ = {*c*_69,001_, *c*_69,002_, …, *c*_72,000_}.
Step 6. The mean ${\bar{c}}_{j}$ and standard deviation $\sigma_{j}$ of the elements in each $b_{j}$ (except outlying values) are calculated, and Equation (2) is used to find the sum $d_{j}$ of these values and is set as the MQF $e_{q}$ = {*d*_1_, *d*_2_, …, *d_j_*, …, *d_p_*}.
  (2) $$d_{j}={\bar{c}}_{j}+\sigma_{j}$$
    Chewing is characterized by greater and continuously changing amplitude. Taking the absolute value of the amount of change and the sum of its mean value and standard deviation produces a larger value for *d_j_* during chewing (greater amplitude indicates a larger mean value and continuous changes in amplitude result in a larger standard deviation).
Step 7. The maximum and minimum MQF are found, and the mean of these two values is set as the threshold. The time during which the MQF is above the threshold represents the mealtime.
    For example, when a subject wears the sensor for two hours per day from 11:00 to 13:00, the MQF *d*_1_ corresponds to 11:00–11:05, the MQF *d*_2_ corresponds to 11:05–11:10, and the MQF *d*_24_ corresponds to 12:55–13:00.

Notice that there exists neither threshold training nor a testing phase in this mealtime estimation algorithm. The threshold for determining whether it is the mealtime or not is always an unknown parameter before executing the algorithm, and it is adaptively calculated at step 7 without prior knowledge about the subject’s personal information. Thus, the threshold differs each time a user wears the sensor regardless of whether or not he or she is the same person. On the other hand, the algorithm is based on the assumption that the measured data will always include mealtimes. A training phase to learn the threshold in advance may be necessary to accurately estimate mealtimes, even for cases in which this prerequisite is not satisfied.

### 2.3. Data Collection Protocol

The following two experiments were performed to confirm if meal times could be measured by the proposed method and if eating could be differentiated from running.

The first experiment was designed to demonstrate the usefulness of the proposed method (sensor and meal time estimation algorithm) for estimating meal times. The subjects of this experiment comprised one woman and six men, all healthy individuals aged 22 years, who were labeled as subjects A to G. These subjects wore the sensor in Figure 3 in their right ear for two hours a day (11:00 to 13:00) and also wore a small PC on their body to record voltages from the sensor and the times of these voltages. The subjects were then asked to spend their daily lives freely without any restrictions in their activities (e.g., eating, conversations, walking, climbing stairs, using a computer, or using a smartphone, removal and insertion of an earphone-type sensor). Once the experiment ended, the subjects were asked to answer a questionnaire about their meal start and end times during the experiment. Moreover, because the sensor used in the experiment had almost the same shape and weight as an ordinary, commercially-available earphone, no discomfort was experienced from the long-term wear of the sensor. The measurement time in this experiment, however, was set at 2 h to minimize subjects’ time constraints. This experiment was performed during lunchtime hours (from 11:00 to 13:00) to accurately evaluate the utility of the meal time estimation algorithm.

The second experiment was designed to investigate if chewing could be differentiated from running. The subjects of this experiment included four fit and healthy men with good dentition and a mean age of 27.8 years (22, 23, 23, 43 years) who were labeled as subjects H to K. Shape changes to the ear canal were measured during chewing and running in these four subjects, and the correlation coefficient of shape changes to the ear canals of the left and right ears was determined. In this experiment, the subjects were asked to wear the sensor presented in Figure 3 in both ears. The subjects also wore a small PC on their body to record the voltages from the sensor and the times of these voltages.

Full explanations of the content and purpose of these experiments were provided to the subjects, who then provided their written informed consent to participate. The personal information of the subjects obtained from these experiments was strictly managed and was not used for any purpose other than that to which the subjects consented.

## 3. Results

The results of the questionnaire and meal time estimation algorithm for subjects A to G are presented in Table 1. Table 1 also shows the ratio of overlap between meal times from the results of the questionnaire and from the meal time estimation algorithm. The ratio of overlap is defined as “the ratio of the logical product of both intervals (interval from the questionnaire and interval from the estimation algorithm) to the logical sum of both intervals”. For example, in the case of subject B, the interval obtained from the questionnaire was from 12:12 to 12:21, whereas the interval obtained from the meal time estimation algorithm was from 12:15 to 12:25. In this case, the logical product of both intervals is an interval from 12:15 to 12:21, and the logical sum between both intervals is an interval from 12:12 to 12:25. The ratio of overlap is therefore 6/13 (=[12:15–12:21]/[12:12–12:25]).

**Figure 8:**
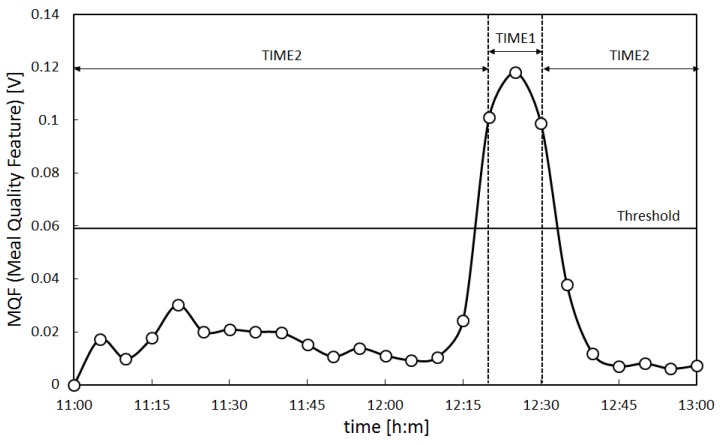
Meal quality feature of subject A. The Meal Quality Feature (MQF) of subject A calculated using the meal time estimation algorithm is presented in the figure. The vertical axis in the figure shows the MQF in volts (V), while the horizontal axis shows the time (11:00 to 13:00) in hours and minutes (h:m). The time during which the MQF is above the threshold is TIME1, while the time during which the MQF is below the threshold is TIME2. TIME1 is the estimated meal time.

In addition, the MQF of subject A calculated using the meal time estimation algorithm is presented in Figure 8. The vertical axis in Figure 8 shows the MQF in volts (V), while the horizontal axis shows the time (11:00 to 13:00) in hours and minutes (h:m). The time during which the MQF is above the threshold is TIME1, while the time during which the MQF is below the threshold is TIME2. TIME1 is the estimated meal time. The mean value for the MQF in TIME1 is Mean1, and the mean value for the MQF in TIME2 is Mean2. From Figure 8, TIME1 of subject A was from 12:20 to 12:30 and Mean1 and Mean2 were 1.06 × 10^−1^ V and 1.49 × 10^2^ V, respectively. TIME1, Mean1, and Mean2 of all subjects were determined by the same method as determined for subject A and are presented in Table 2. When the actual meal times of all subjects obtained by the questionnaire were compared with the estimated meal times (TIME1) obtained by the algorithm, the differences for each subject fell within a range of five minutes. From Table 2, it is evident that the largest Mean1 was in subject A, at 1.06 × 10^−1^ V, while the smallest Mean1 was in subject B, at 1.31 × 10^−2^ V, revealing individual differences in Mean1 values. These results indicate that despite individual differences among subjects, the variability between the questionnaire results and estimated results fell within the pre-set range of accuracy (within five minutes) for all subjects, and the meal time estimation algorithm was capable of correctly estimating meal times within the scope of the experiment.

**Figure 9:**
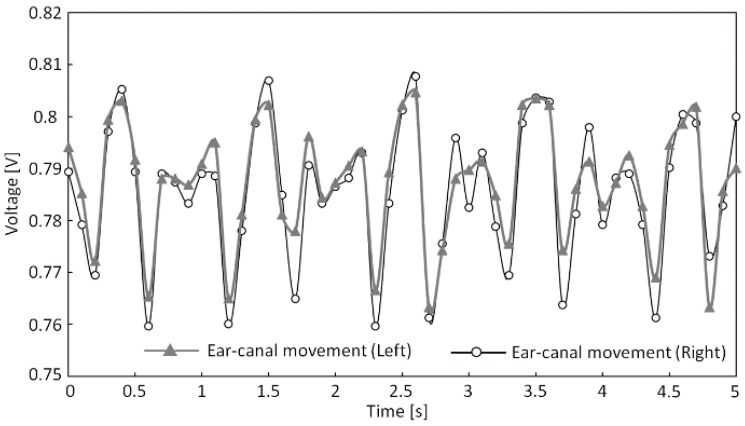
Right and left ear canal movement during running (subject H).

**Figure 10:**
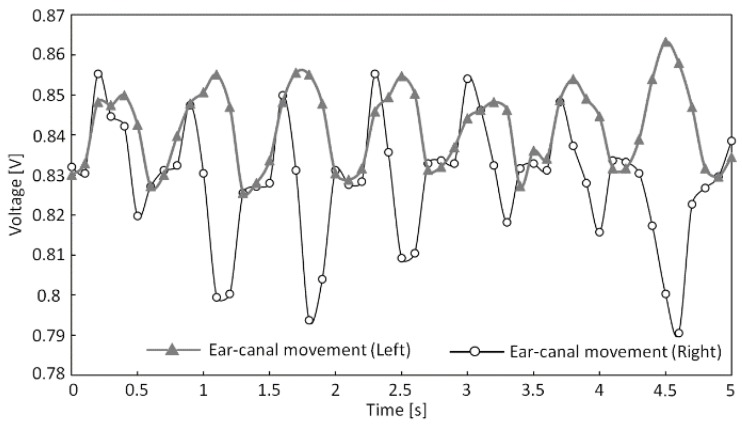
Right and left ear canal movement during chewing (subject H).

We next determined Pearson’s product–moment correlation coefficient (hereafter referred to as “correlation coefficient”) from the measured shape changes to the left and right ear canals during running and chewing gum in subjects H to K, which are presented in Table 3. In addition, Figure 9 and Figure 10 show the measured shape changes to the left and right ear canals during running and chewing for subject H. The vertical axes in Figure 9 and Figure 10 show the output voltage from the sensor in volts (V), while the horizontal axes show the time in seconds. In Table 3, data for subjects H to K are listed, and the largest correlation coefficient *r* was 0.941 for subject J and the smallest coefficient *r* was 0.741 for subject I during running. In contrast, during gum chewing, the largest correlation coefficient *r* was 0.384 in subject J and the smallest coefficient *r* was 0.175 in subject K. Figure 9 and Figure 10 also show that the left and right measured values are almost synchronized during running, whereas they are not synchronized during chewing. Therefore, Table 3 and Figure 9 and Figure 10 reveal a strong correlation in the measured results for the left and right ear canals during running but not during chewing. A clear difference was observed in the correlation coefficients of the measured results for the left and right ear canals during running and chewing within the scope of this experiment.

## 4. Discussion

Based on the experimental results, we first discuss whether the proposed method can estimate meal times (food intake) and differentiate eating (chewing) from running.

We can conclude that this sensor and algorithm can be used to estimate meal times within the scope of our experimental results. This is because the difference between the actual and estimated meal times (TIME1) fell within five minutes for all subjects in our experimental results and Mean2 was less than 50% of Mean1. This demonstrates that meal times can be estimated regardless of individual differences in the shape changes to the subjects’ ear canals using this sensor and algorithm. An error of up to five minutes, nevertheless, exists between the actual meal times and those estimated using this algorithm. This error is valid because MQF is defined as five minutes in the algorithm used in this study. If it was necessary to estimate the meal times to a higher degree of accuracy less than five minutes, a solution could be found by changing the time to calculate the MQF in accordance with the required accuracy. However, it is not theoretically possible to reduce the error to zero because this algorithm uses statistical processing.

Next, we discuss the impact of individual differences in shape changes to the ear canal on the meal time estimation results. All subjects had large MQF values for the actual meal times; however, these values differed for each subject. When the experimental results for Mean1 were compared between the seven subjects, we observed the largest Mean1 in subject A, at 1.06 × 10^−1^ V, and the smallest Mean1 in subject B, at 1.31 × 10^−2^ V. The difference in these values can be indicative of the effect of individual differences in shape changes to the ear canal. The MQF is calculated from the sum of the mean and standard deviation of the amount of change based on the output voltage of the sensor. When the MQF is high, the mean and standard deviation for the amount of change are also large, which in turn indicate a substantial change in the distance between the sensor and eardrum. Thus, the MQF is large when the subject’s ear canal movement is large. In the case of this experiment, subject A exhibited the largest shape change to the ear canal, while subject B exhibited the smallest. Shape changes to the ear canal differ according to each individual, and these individual differences directly influence the MQF. However, the MQF of TIME1 was more than two-times larger than that of TIME2 for all subjects, indicating a clear difference between TIME1 and TIME2. These results suggest that meal times can be estimated within the range of error by setting a threshold based on the maximum and minimum MQF values of each subject. This is also performed in this algorithm, without being influenced by individual differences in shape changes to the ear canal. In the future, we aim to improve the accuracy of this algorithm by continuing to study meal time windows (e.g., meal times ranging from 5 min to 1 min).

We next discuss if chewing (eating) can be differentiated from running. We found that this sensor can be used to differentiate eating from running within the scope of our experimental results. This is because the measured results for the left and right ear canals during running strongly correlated with each other, whereas the results during chewing did not. If these activities are categorized based on correlation coefficients, they can be distinguished from each other. In particular, the measured waveforms of the left and right ear canals during running are similar, as shown in Figure 6, whereas measurements during chewing are not similar between the left and right ear canals. Table 3 shows that the correlation coefficient *r* during running was 0.941 at its highest and 0.741 at its lowest. Meanwhile, the correlation coefficient *r* during chewing was 0.384 at its highest and 0.175 at its lowest. Therefore, these findings suggest that this is a viable method for using a single (fixed) threshold to differentiate eating from running, in marked contrast with the mealtime estimation algorithm, which adaptively varies the threshold every time a user wears the sensor. For example, running is identified when the correlation coefficient *r* calculated from the measured results of shape changes to the left and right ear canals exhibits a strong correlation equal to or greater than 0.7. In contrast, eating is identified when the correlation coefficient *r* exhibits no correlation or a weak correlation equal to or lower than 0.4 and when the meal time estimation algorithm identifies eating.

Now, we further discuss running and chewing based on the data presented in Figure 6 and Figure 7. The running waveform in Figure 6 shows that the left and right ear canal waveforms are similar during running. On the other hand, the chewing waveform in Figure 7 reveals that the left and right ear canal waveforms differ during chewing. This indicates that during running, the same waveform resulting from running is superimposed onto the measured results for the left and right ear canals. The reason for this superimposition of the waveform onto the measured results is due to vibrations generated by the foot. The vibrations that occur when the foot makes contact with the ground are transmitted to the ear via the body and are consequently measured by the sensor. Running involves one foot at a time making contact with the ground; however, the same running waveform is superimposed onto the measured results for shape changes to the left and right ear canals. This is because the transmission pathways of the vibrations generated by the feet are the same. The pathways by which the vibrations that occur when the feet make contact with the ground differ in how they are transmitted between the left and right feet from the soles of the feet up to the hips. However, from the hips to the lower back, chest, neck, and head, vibrations arrive via the same pathway, regardless of the foot from which they originated. This is why transmitted vibrations are not biased toward any side and why running leads to the same results being superimposed onto the measured results for shape changes to the left and right ear canals. Therefore, the measured results for shape changes to the left and right ear canals associated with running are similar. In contrast, the measured results for shape changes to the left and right ear canals associated with chewing are not the same, as evident in Figure 7. Chewing causes the temporal muscles to expand and contract, which results in movement of the ear canal. If shape changes to the left and right ear canals associated with chewing were the same, the temporal muscles would expand and contract at the same time and in the same manner. Furthermore, if the left and right temporal muscles were to expand and contract at the same time and in the same way as a result of chewing, the jaw would move precisely up and down. However, the actual jaw movement during chewing is biased toward either the left or right. Because the left and right temporal muscles do not expand and contract at the same time or in the same way, shape changes to the ear canal associated with chewing differ between the left and right sides. Based on the above findings, we intend to estimate the amount of movement by analyzing the waveforms during running. In addition to estimating the amount of movement, we are currently conducting pulse measurements within the ear canal by using our proposed method and the same type of optical sensor. If we can estimate the amount of movement and improve the accuracy of pulse measurements, we can create a device capable of supporting a healthy lifestyle among users regarding exercise and diet by simply obtaining measurements from just one of the optical sensors inserted into the left or right ear canals.

## 5. Conclusions

In this paper, we described a method for estimating meal times based on temporal changes in the amount of light received by a small optical sensor composed of an LED and a phototransistor inserted in the right ear canal. The device shines light toward the inner ear canal and receives light reflected back by the ear canal. An experiment performed using seven subjects was successful in estimating meal times in all subjects within a set range of error. Furthermore, an analysis of the measured values during running and chewing obtained from the same sensor inserted into the left and right ear canals of four subjects revealed that measurements from the left and right ear canals were strongly correlated during running, but not correlated during chewing. These findings allow running and chewing to be differentiated based on correlation coefficients.

In the future, we intend to develop a method for estimating the amount of movement of the body by analyzing waveforms during running. In addition to improving our meal time estimation algorithm and continuing to investigate methods for differentiating running and chewing, we aim to conduct further experiments using larger subject samples. We also hope to establish a method for measuring the pulse within the ear canal using our proposed method and the same type of optical sensor. Among the research on detecting chewing sounds via the ear, there is a study that used a microphone worn in the ear to sense chewing sounds transmitted through the bone [24]. More accurate meal time measurements might be achieved by combining the results of our study with those from the study that used a microphone. By combining all these research results, we aim to develop a device capable of supporting a healthy lifestyle of users regarding both exercise and diet.

## Figures and Tables

**Table 1:** Experimental results of meal time measurements. The results of the questionnaire and meal time estimation algorithm for subjects A to G are presented in Table 1. Table 1 also shows the ratio of overlap between meal times from the results of the questionnaire and the meal time estimation algorithm. The ratio of overlap is defined as “the ratio of the logical product of both intervals (interval from the questionnaire and interval from the estimation algorithm) to the logical sum of both intervals”.

Subject	Questionnaire Results (h:m)	Estimated Results (h:m)	% Overlap
A	12:14–12:30	12:20–12:30	5/8
B	12:12–12:21	12:15–12:25	6/13
C	12:10–12:18	12:15–12:20	3/10
D	12:06–12:20	12:10–12:20	5/7
E	12:20–12:37	12:25–12:40	3/5
F	12:35–12:52	12:40–12:55	3/5
G	12:24–12:29	12:25–12:30	2/3

**Table 2:** Experimental results. The mean value for the MQF in TIME1 is Mean1, and the mean value for the MQF in TIME2 is Mean2.

Subject	Mean1 (V)	Mean2 (V)
A	$1.06\times 10^{-1}$	$1.49\times 10^{-2}$
B	$1.31\times 10^{-2}$	$5.42\times 10^{-3}$
C	$8.21\times 10^{-2}$	$1.39\times 10^{-3}$
D	$3.15\times 10^{-2}$	$8.20\times 10^{-3}$
E	$3.05\times 10^{-2}$	$9.05\times 10^{-3}$
F	$5.82\times 10^{-2}$	$6.90\times 10^{-3}$
G	$7.20\times 10^{-2}$	$9.90\times 10^{-3}$

**Table 3:** Correlation coefficients of shape changes to the left and right ear canals (similarity of shape changes to the left and right ear canals).

Subject	Running	Chewing
H	0.796	0.221
I	0.741	0.359
J	0.941	0.384
K	0.757	0.175
